# Investigating the potential added value of [^18^F]FDG-PET/CT in long COVID patients with persistent symptoms: a proof of concept study

**DOI:** 10.1097/MNM.0000000000001689

**Published:** 2023-03-24

**Authors:** Linda L. Chen, Alina van de Burgt, Frits Smit, Rowena S. Audhoe, Sandra M. de Boer, Floris H.P. van Velden, Lioe-Fee de Geus-Oei

**Affiliations:** a Department of Nuclear Medicine, Alrijne Hospital, Leiderdorp; b Department of Technical Medicine, Faculty of 3mE, Delft University of Technology, Delft; c Department of Radiology, Section of Nuclear Medicine, Leiden University Medical Center (LUMC), Leiden; d Department of Radiation Therapy, Erasmus University Medical Center, Rotterdam; e Biomedical Photonic Imaging Group, Twente University, Enschede; f Department of Radiation Science & Technology, Delft University of Technology, Delft, The Netherlands

**Keywords:** imaging, infection, inflammation, severe acute respiratory syndrome coronavirus 2, vasculitis

## Abstract

Since the end of 2019, the coronavirus disease 2019 (COVID-19) virus has infected millions of people, of whom a significant group suffers from sequelae from COVID-19, termed long COVID. As more and more patients emerge with long COVID who have symptoms of fatigue, myalgia and joint pain, we must examine potential biomarkers to find quantifiable parameters to define the underlying mechanisms and enable response monitoring. The aim of this study is to investigate the potential added value of [^18^F]FDG-PET/computed tomography (CT) for this group of long COVID patients.

**Methods:**

For this proof of concept study, we evaluated [^18^F]FDG-PET/CT scans of long COVID patients and controls. Two analyses were performed: semi-quantitative analysis using target-to-background ratios (TBRs) in 24 targets and total vascular score (TVS) assessed by two independent nuclear medicine physicians. Mann–Whitney *U*-test was performed to find significant differences between the two groups.

**Results:**

Thirteen patients were included in the long COVID group and 25 patients were included in the control group. No significant differences (*P* < 0.05) were found between the long COVID group and the control group in the TBR or TVS assessment.

**Conclusion:**

As we found no quantitative difference in the TBR or TVS between long COVID patients and controls, we are unable to prove that [^18^F]FDG is of added value for long COVID patients with symptoms of myalgia or joint pain. Prospective cohort studies are necessary to understand the underlying mechanisms of long COVID.

## Introduction

Since the end of 2019, the severe acute respiratory syndrome coronavirus 2 (SARS-CoV-2) virus has been disrupting lives globally despite extensive efforts to contain the virus [1]. The most common symptoms of acute coronavirus disease 2019 (COVID-19) are fever, dry cough and fatigue, albeit the disease expression is highly heterogeneous [1–4]. About 80% of the patients experience mild to moderate disease, whilst 5% develop critical illness [5,6]. Moreover, the majority of patients develop sequelae after recovering from the acute SARS-CoV-2 infection that lasts for weeks to months [7]. This is called long COVID, or post-COVID syndrome [6,8,9]. Symptoms associated with long COVID include fatigue, dyspnoea, poor memory, hair loss, joint pain, attention disorder and myalgia, although this disease expression is also heterogeneous [4,10]. The onset of arthritis and vasculitis has also been reported in long COVID patients, and there is a growing recognition that COVID-19 is a vascular disease that leads to an escalating cascade of inflammatory pathways [11–14].

Long COVID patients are often PCR-negative and show no radiological or biochemical abnormalities. The lag of clinical recovery can be exasperating, which causes mental problems on top of physical problems [6]. As the primary focus of the pandemic was to investigate the optimal treatment for acute COVID-19 patients and deal with the latest mutations of SARS-CoV-2, optimizing the rehabilitation of long COVID patients lagged. As a result, clear guidelines on the optimal treatment for long COVID patients are lacking [4].

As more and more patients with COVID in the medical history are emerging with vague symptoms, for example, fatigue, joint pain and myalgia similar to vasculitis, sarcoidosis and polymyalgia rheumatica, there is a need for (imaging) biomarkers to find quantifiable parameters in order to define the underlying mechanisms. As a result, this would enable evaluation of disease activity and treatment response monitoring. [^18^F]FDG-PET/computed tomography (CT) can potentially be of added value in this process, as [^18^F]FDG-PET/CT is able to determine localized metabolic activity, including infection, inflammation and malignancies [15]. Abnormal [^18^F]FDG-PET/CT scans in long COVID patients have been observed in earlier studies, albeit no study has been able to discern a typical visual [^18^F]FDG-uptake pattern in long COVID patients yet, which shows the need for further investigation [16–18]. This study aims to investigate the potential added value of [^18^F]FDG-PET/CT for long COVID patients with persistent symptoms such as myalgia, joint pain and fatigue, reminiscent of vasculitis, sarcoidosis and polymyalgia rheumatica.

## Methods

### Study design and population

To investigate the potential added value of [^18^F]FDG-PET/CT for long COVID patients with persistent symptoms, we performed a retrospective proof of concept study to qualitatively and quantitatively compare [^18^F]FDG-PET/CT scans of long COVID patients and controls.

For the long COVID patient group, we included patients from our long COVID outpatient clinic who presented with symptoms of myalgia or joint pain, reminiscent of vasculitis, polymyalgia rheumatica or sarcoidosis, for whom an [^18^F]FDG-PET/CT scan was performed between May 2021 and October 2021. This study was retrospective and approval by the medical ethics committee was therefore not required according to the Dutch law. Nevertheless, written informed consent was obtained from all participants.

For the control group, we included patients who either had a malignancy in the past for which they were exclusively surgically curatively treated and for whom a routine [^18^F]FDG-PET/CT follow-up scan was performed, excluding recurrent/residual disease; or received an [^18^F]FDG-PET/CT scan for a suspected malignancy or aetiology of unknown origin, which did not show any disease. Moreover, we exclusively included [^18^F]FDG-PET/CT scans from June 2019 until October 2021, as the hospital acquired a new PET/CT scanner in June 2019. We did not include patients in the control group who had received systemic oncological treatment or radiotherapy in the past or had inflammatory diseases such as sarcoidosis, vasculitis, rheumatic diseases or COVID-19 in their medical history.

Baseline information was gathered from the electronic health records consisting of sex, age, BMI, pre-PET glycaemia, administered [^18^F]FDG activity, interval time between [^18^F]FDG administration and scan acquisition and medicine use. Differences in age, BMI, pre-PET glycaemia, administered [^18^F]FDG activity and interval time between [^18^F]FDG administration and scan acquisition were investigated with an unpaired two-tailed Student’s *t*-test and differences in sex using a chi-squared test. We considered *P* < 0.05 to be significant.

### Data collection

We anonymized patient data and recorded these in a database. Whole-body [^18^F]FDG-PET/CT was performed for long COVID patients on the 5-Ring Discovery MI PET/CT (GE Healthcare, Chicago, Illinois, USA) [19]. Control [^18^F]FDG-PET/CT scans were acquired as whole-body images if available and as torso (mid-thigh to skull base) images if no whole-body images were available. Data acquisition was performed approximately 60 min after intravenous [^18^F]FDG administration (1.5 MBq per kg bodyweight if BMI < 30, 2.1 MBq per kilogram bodyweight if BMI > 30). Of note, due to use of a high sensitive PET/CT system this dose is lower than the standard 3.0 MBq per kilogram bodyweight. An emission scan was obtained using multiple bed positions (50% overlap between bed positions, 75 s per bed position) [19]. Time-of-flight PET data were reconstructed using the point spread function and CT-based attenuation correction (120 kV, smart mA modulations with a noise index of 49.5 and an mA ranging from 15 to 550, 0.5 s rotation time). Body-weighted standardized uptake values (SUVs) were obtained using Sectra IDS7 (Sectra AB, Linköping, Sweden) (PACS).

### Data analysis

**Fig. 1:**
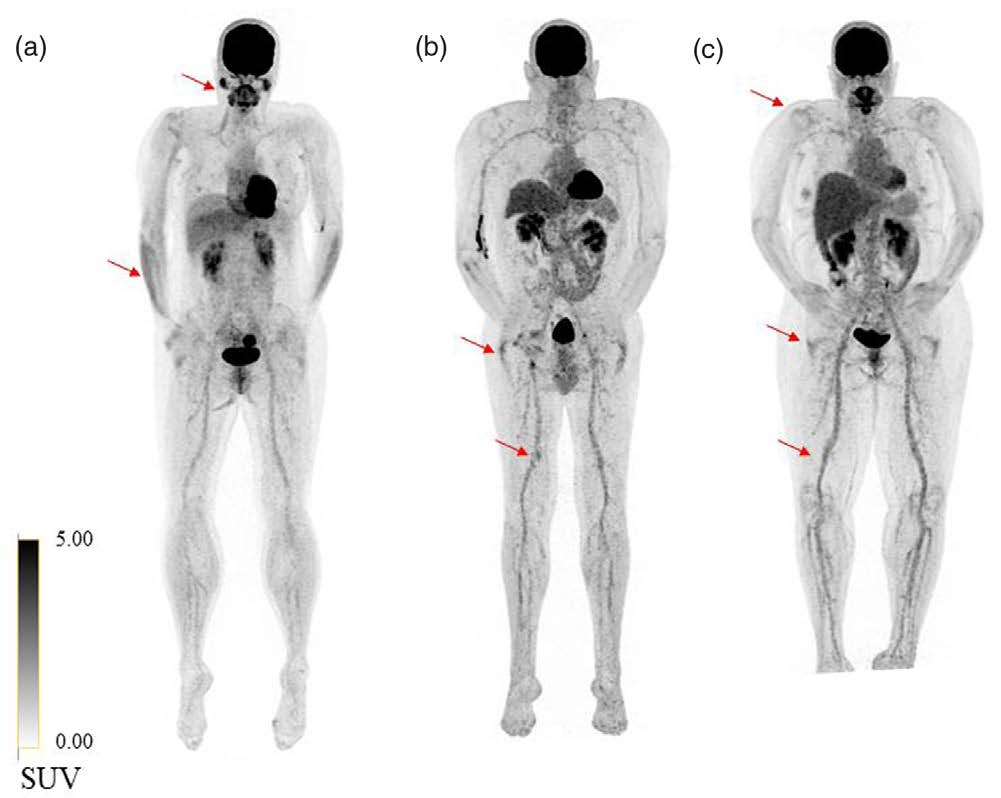
Examples of three long COVID patients with persistent symptoms of fatigue, myalgia or joint pain. (a) High to moderate [^18^F]FDG-uptake in the m. brachioradialis and parotid glands (red arrows). (b and c) Low to moderate [^18^F]FDG-uptake in joints and vessels in the lower extremities (red arrows). COVID, coronavirus disease.

To compare long COVID patients with controls, a semi-quantitative analysis was performed by determining the target-to-background ratio (TBR) according to the nine research targets described in the European Association for Nuclear Medicine (EANM) recommendations for [^18^F]FDG-PET/CT imaging in large vessel vasculitis (LVV) and polymyalgia rheumatica to account for the variability of SUVs: the carotid, subclavia, axillary, vertebral and pulmonary arteries, the ascending, descending, and abdominal aorta and the aortic arch [20–23]. Additional targets consisted of the parotid glands, external iliac arteries, femoral arteries, tibial arteries, the liver and the brachioradialis muscle. The background was calculated as the average SUV in the vena cava inferior and vena cava superior (Fig. 1).

In order to assess the difference in overall [^18^F]FDG-uptake between the long COVID group and control group, we used the total vascular score (TVS), as this takes heterogeneity within groups into account. The TVS was determined using the seven clinical targets described in the EANM recommendations for [^18^F]FDG-PET/CT imaging in LVV and polymyalgia rheumatica: thoracic aorta, abdominal aorta, subclavian arteries, axillary arteries, carotid arteries, iliac arteries and femoral arteries [22]. Additional targets consisted of the parotid glands, shoulder girdle and hip girdle (analysis of 10 targets, performed on all patients). Additionally, the tibial arteries, lower arm muscles and hands were assessed if visible on the scan (analysis of 13 targets, performed on patients with total body scans). A standardized 0–3 grading system was used to assess all targets, and was defined as follows: 0 = physiological [^18^F]FDG-uptake; 1 = minimally heightened [^18^F]FDG-uptake (<mediastinum), 2 = clearly increased [^18^F]FDG-uptake (≥mediastinum and <liver), 3 = very marked [^18^F]FDG-uptake (≥liver). The 10 or 13 targets per patient were assessed independently by two experienced nuclear medicine physicians (S.M.d.B., R.S.A.) who were blinded. The TVS was calculated as the sum of all target scores.

### Statistical analysis

For the semi-quantitative analysis, the mean, median, SD and range were calculated for every TBR and subject (Supplementary Table A, Supplemental digital content 1, http://links.lww.com/NMC/A244). We performed the Mann–Whitney *U*-test to assess the difference between the long COVID group and control group for each target and applied Bonferroni correction for multiple testing [24]. We considered a *P* value < 0.05 to be statistically significant.

For the TVS analysis, the interclass correlation was determined to assess intra-observer similarity. A threshold of interclass correlation < 0.75 was agreed upon to analyse the two observers separately. A separate Mann–Whitney *U*-test was performed for all subjects using 10 targets (maximum number of subjects) and subjects with 13 targets (maximum number of targets). We considered a *P* value < 0.05 to be statistically significant. We used Excel (Version 2109; Microsoft, Albuquerque, New Mexico, USA) for data collection and Matlab (version R2019b; MathWorks, Natick, Massachusetts, USA) to perform the statistical tests.

## Results

### Patient characteristics

Thirteen patients were included in the long COVID group and 25 patients were included in the control group (follow-up after melanoma *n* = 10, disproved suspicion of malignancy *n* = 8, follow-up after mammary carcinoma *n* = 2, follow-up after colon carcinoma *n *= 2, other indications *n* = 3). The long COVID group was on average significantly younger than the control group (47.2 ± 13.09 vs 58 ± 15.62, respectively, *P* = 0.017) and consisted of less males (38.5% vs 46.2%, respectively, *P* = 0.010). Table 1 summarizes the baseline characteristics of both groups.

**Table 1:** Baseline subject characteristics

	Long COVID (*n* = 13)	Controls (*n* = 25)	*P* value
Sex (male, %)	5 (38.5%)	11 (44%)	0.016
Age (mean, std, year)	47.2 (13.09)	57.3 (15.5)	0.024
BMI status (mean, std, kg/m^2^)	24.11 (4.09)	24.62 (3.89)	0.89
Pre-PET glycaemia (mmol/L)	5.33 (1.31)	5.96 (1.91)	0.23
Administered [^18^F]FDG activity (MBq)	123.87 (34.35)	132.93 (38.46)	0.58
Interval time between [^18^F]FDG injection and image acquisition (min)	51.38 (8.49)	48.52 (5.96)	0.44
Symptoms			
Fatigue	13	0	
Pain	6	0	
Dyspnoea	4	0	
Loss of strength	3	0	
Comorbidities (*n*)			
No	2	4	
Diabetes	1	5	
Hypertension	2	2	
Chronic respiratory disease	3	4	
Concomitant medications (*n*)			
None	5	9	
Beta-blockers	0	4	
Calcium antagonists	0	3	
Sartans	0	1	
ACE inhibitors	1	2	
Diuretics	0	1	
Oral anticoagulants	1	2	
Antiplatelet drugs	0	1	
Hypoglycaemic drugs	1	3	
Corticosteroids	2	3	
Statins	1	4	
NSAIDs	3	3	
Benzodiazepines	1	2	
Proton pump inhibitors	4	5	
Bronchodilators	2	2	

ACE, angiotensin-converting enzyme; COVID, coronavirus disease.

### [^18^F]FDG-PET/CT parameters

We found no significant differences between the long COVID and control group with regards to pre-PET glycaemia (5.33 ± 1.31 vs 5.96 ± 1.91 mmol/L, *P* = 0.23), administered [^18^F]FDG activity (123.87 ± 34.35 vs 132.93 ± 38.46 MBq, *P* = 0.58) and interval time between [^18^F]FDG injection and image acquisition [51.38 ± 8.49 (range 41–74) vs 48.52 ± 5.96 (range 40–68) min, *P* = 0.44] (Table 1).

### Clinical data

Long COVID patients in this study reported symptoms of fatigue, dyspnoea, concentration problems, myalgia, asthenia and low mood. The severity of the COVID-19 infection ranged from mild to severe. Symptoms during infection included fatigue, dyspnoea, cough, fever, myalgia and loss of taste or smell. The time interval between COVID-19 infection and [^18^F]FDG-PET/CT scan was 9.0 ± 4.4 months. None of the 13 long COVID patients were admitted to the hospital for the COVID-19 infection.

### Target-to-background ratio [^18^F]FDG-PET/CT analysis

Table 2 shows the results of the semi-quantitative analysis of all targets. No targets differed significantly between the long COVID group compared to the control group. Increased [^18^F]FDG-uptake of the parotid glands was observed in 6/13 long COVID patients and 6/26 control patients (TBR_parotid_ gland left = 1.34 vs 1.02, respectively, *P* = 5.52 and TBR_parotid_ gland right = 1.37 vs 1.01, respectively, *P* = 3.35). We also observed a higher [^18^F]FDG-uptake in the liver in the long COVID group than in the control group (TBR_liver_ = 1.47 vs 1.34, respectively, *P* = 0.18).

**Table 2:** Results of semi-quantitative analysis in the long coronavirus disease group and control group

	TBR mean (std)		
	Long COVID (*n* = 13)	Controls (*n* = 25)	*P* value
a. carotis communis sinistra (mean, std)	1.06 (0.16)	0.99 (0.25)	4.97
a. carotis communis dextra	1.04 (0.18)	1.03 (0.18)	21.07
a. subclavia sinistra	0.91 (0.16)	0.94 (0.15)	11.50
a. subclavia dextra	0.92 (0.18)	0.98 (0.22)	5.24
a. axillaris sinistra	0.88 (0.29)	0.92 (0.12)	16.00
a. axillaris dextra	0.79 (0.30)	0.94 (0.15)	0.87
a. vertebralis sinistra	0.90 (0.17)	0.85 (0.15)	8.54
a. vertebralis dextra	0.86 (0.18)	0.87 (0.23)	24.00
Ascending aorta	1.10 (0.08)	1.06 (0.06)	8.35
Aortic arch	1.07 (0.08)	1.06 (0.11)	17.09
Pulmonary arteries	1.09 (0.11)	1.08 (0.08)	6.76
Descending aorta	1.06 (0.07)	1.07 (0.09)	23.12
Abdominal aorta	1.10 (0.19)	1.09 (0.12)	16.00
Glandula parotis sinistra	1.34 (0.75)	1.02 (0.41)	5.52
Glandula parotis dextra	1.37 (0.78)	1.01 (0.43)	3.35
a. iliaca externa sinistra	1.03 (0.24)	1.04 (0.19)	13.41
a. iliaca externa dextra	0.95 (0.22)	1.05 (0.16)	3.35
a. femoralis sinistra	1.04 (0.25)	0.93 (0.19)	9.75
a. femoralis dextra	1.00 (0.20)	0.98 (0.26)	18.20
a. tibialis sinistra	1.02 (0.29)	0.93 (0.15)	13.50
a. tibialis dextra	0.99 (0.29)	0.96 (0.18)	11.69
Liver	1.47 (0.12)	1.34 (0.11)	0.18
m. brachioradialis sinistra	0.49 (0.14)	0.48 (0.23)	14.59
m. brachioradialis dextra	0.49 (0.16)	0.47 (0.11)	18.20

COVID, coronavirus disease; TBR, target-to-background ratio.

### Total vascular score [^18^F]FDG-PET/CT analysis

**Fig. 2:**
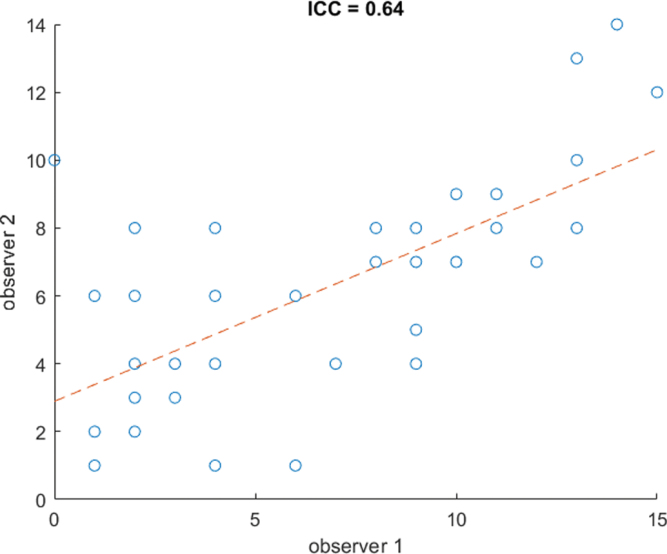
Scatterplot of TVS between the two observers. ICC, intraclass correlation coefficient; TVS, total vascular score.

Moderate agreement was obtained between the two observers (interclass correlation = 0.65, *P* < 0.001), meaning we performed separate analyses for the two observers (Fig. 2). We found a mean TVS of 3.00 ± 2.42 (observer 1) and 4.46 ± 2.07 (observer 2) in the long COVID group versus 3.60 ± 2.45 (observer 1) and 5.12 ± 2.62 (observer 2) in the control group in the analysis of 10 targets (long COVID group *n* = 13, control group *n* = 25), as is shown in Table 3. No observer reported a significant difference between the two groups (*P* = 0.53 and *P* = 0.52, respectively). We found a mean TVS of 8 ± 4.42 (observer 1) and 7.08 ± 3.66 (observer 2) in the long COVID group versus 9.56 ± 2.24 (observer 1) and 6.78 ± 3.35 (observer 2) in the control group in the analysis of 13 targets (long COVID group *n* = 13, control group *n* = 9), as is shown in Table 4. This yielded no significant differences for both observers (*P* = 0.37 and *P* = 0.92).

**Table 3:** Total vascular score in the long coronavirus disease group (*n* = 13) and control group (*n* = 26), 10 targets

	TVS mean (std)	
	Observer 1	Observer 2
TVS long COVID group	3 (2.42)	4.46 (2.07)
TVS control group	3.60 (2.45)	5.12 (2.62)
*P* value	0.53	0.52

COVID, coronavirus disease; TVS, total vascular score.

**Table 4:** Total vascular score in the long coronavirus disease group (*n* = 13) and control group (*n* = 9), 13 targets

	TVS mean (std)	
	Observer 1	Observer 2
TVS long COVID	8.00 (4.42)	7.08 (3.66)
TVS control group	9.56 (2.24)	6.78 (3.35)
*P* value	0.37	0.47

COVID, coronavirus disease; TVS, total vascular score.

## Discussion

In this proof of concept study, we investigated the potential added value of [^18^F]FDG-PET/CT for long COVID patients with persistent symptoms. No significant differences were found between the long COVID group and the control group in the semi-qualitative analysis and TVS; however, although several long COVID patients showed higher uptake in one or more of these targets, we were unable to identify a general pattern.

In a similar study performed by Sollini *et al*., a significant difference was found between the long COVID group and the control group in several targets, including the right femoral artery, the ascending aorta, the aortic arch and the descending aorta [16]; however, no correction for multiple testing was performed and if applied, no significant differences would have been found. In the current study, the Bonferroni correction for multiple testing was used to prevent false positive findings [25]. If no Bonferroni correction had been applied, we would have found significant differences in the liver and the right axillary arteries between the long COVID group and control group (Supplementary Table A, Supplemental digital content 1, http://links.lww.com/NMC/A244), which illustrates the similarity of results between Sollini *et al*. and the current study; however, it should be noted that long COVID presentation could be heterogeneous in nature, as other rheumatic diseases such as polymyalgia rheumatica also show high heterogeneity across patients on [^18^F]FDG-PET/CT scans [26,27]. Furthermore, similar studies also found heterogeneous differences between long COVID patients and controls [17,18].

The liver has been reported as a COVID-19 target organ [28,29] and recent studies suggest that the liver might be still inflamed in long COVID [17,30]. This possibly explains the higher liver uptake in the long COVID group compared to the control group.

The results of this study should be considered alongside certain limitations. Firstly, the study population consisted of 13 long COVID patients, limiting the power of the study. The control group could not be matched for age and sex to the long COVID group due to limitations in our database. Due to the large heterogeneity of [^18^F]FDG-uptake within both groups, the TBR distributions overlapped between the two groups. This was an important cause for the high *P* values we found in the semi-qualitative and TVS analyses.

Another limitation is that the image reconstruction was not European Association of Nuclear Medicine (EARL) compliant because EARL specifications were not yet applied at the time of acquisition, which might have resulted in increased SUV variability [31]. Moreover, the time interval between [^18^F]FDG injection and image acquisition was lower than 60 min in both groups due to logistic reasons, which deviates from the EANM recommendation for LVV and polymyalgia rheumatica, which Sollini *et al.* did adhere to [16,22]. This may have had an impact on the results, although no guidelines on the recommended time interval between [^18^F]FDG injection and image acquisition have been published for long COVID patients and there were no significant differences in interval time between the two groups.

Moreover, the scanning window (whole-body imaging vs torso imaging) should be consistent for all subjects in order to be able to assess all locations [32]. Future studies should also consider specifying and quantifying the location of pain per patient.

It should also be noted that all measurements were manually performed and thus prone to errors [31]. Nonetheless, the nuclear medicine physicians (S.M.d.B., R.S.A.) had a minimum of 5 years of experience and measurements were performed carefully, minimizing the number of random errors.

Little is known about long COVID and patients’ management is still inconsistent due to a lack of clinical practice guidelines. Furthermore, [^18^F]FDG-PET/CT data in long COVID patients were limited. Although our retrospective proof of concept study concerns only a small study population, we believe that our findings exhibit the complexity of the disease and add to the knowledge of the application of [^18^F]FDG-PET/CT in long COVID.

In summary, we found no quantitative difference in the TBR or TVS between long COVID patients and controls. On the basis of our results, we are unable to prove that [^18^F]FDG-PET/CT scans are of added value for long COVID patients with symptoms of myalgia or joint pain, reminiscent of vasculitis and polymyalgia rheumatica. To gain more insight into the underlying mechanisms of long COVID, prospective cohort studies are necessary.

## Acknowledgements

We appreciate the specialists of the multidisciplinary team for the long COVID at the Alrijne hospital in Leiderdorp for the numerous helpful discussions regarding individual patients and the most important considerations in the treatment of these patients. No funding was received for conducting this study.

### Conflicts of interest

There are no conflicts of interest.

## Supplementary Material


