# Supplementary material for: Investigating the potential added value of [18F]FDG-PET/CT in long COVID patients with persistent symptoms: a proof of concept study
Source: Nucl Med Commun. 2023 Mar 24;44(6):495–501. doi: 10.1097/MNM.0000000000001689 (PMC10171287; doi:10.1097/MNM.0000000000001689)
Supplement: Supplementary file 1 [file nmc-44-495-s001.pdf]

Supplementary table A. Results of semi-quantitative analysis in the long COVID group and control group with and without Bonferroni correction.

	Long COVID (n=13)	Controls (n=25)	p-value, Bonferroni corrected	p-value, without Bonferroni correction
a. carotis communis sinistra TBR				
- Mean	**1.06**	**0.99**	4.97	**0.21**
- Median	1.04	0.99		
- Standard deviation	0.16	0.25		
- Range	0.69-1.38	0.48-1.82		
a. carotis communis dextra TBR				
- Mean	**1.04**	**1.03**	21.07	**0.88**
- Median	0.98	1.00		
- Standard deviation	0.18	0.18		
- Range	0.81-1.56	0.72-1.42		
a. subclavia sinistra TBR				
- Mean	**0.91**	**0.94**	11.50	**0.48**
- Median	0.84	0.92		
- Standard deviation	0.16	0.15		
- Range	0.73-1.22	0.72-1.37		
a. subclavia dextra TBR				
- Mean	**0.92**	**0.98**	**5.24**	**0.22**
- Median	0.85	0.95		
- Standard deviation	0.18	0.22		

- Range	0.71-1.33	0.69-1.90		
a. axillaris sinistra TBR				
- Mean	**0.88**	**0.92**	**16.00**	**0.67**
- Median	0.88	0.90		
- Standard deviation	0.29	0.12		
- Range	0.43-1.37	0.62-1.21		
a. axillaris dextra TBR				
- Mean	**0.79**	**0.94**	**0.87**	**0.04**
- Median	0.79	0.94		
- Standard deviation	0.30	0.15		
- Range	0.34-1.35	0.67-1.21		
a. vertebralis sinistra TBR				
- Mean	**0.90**	**0.85**	**8.54**	**0.36**
- Median	0.85	0.82		
- Standard deviation	0.17	0.15		
- Range	0.62-1.30	0.59-1.23		
a. vertebralis dextra TBR				
- Mean	**0.86**	**0.87**	**24.00**	**1.00**
- Median	0.81	0.80		
- Standard deviation	0.18	0.23		
- Range	0.67-1.39	0.54-1.53		
Ascending aorta TBR				
- Mean	**1.10**	**1.06**	**8.35**	**0.35**
- Median	1.09	1.06		
- Standard deviation	0.08	0.06		
- Range	0.97-1.27	0.94-1.23		

Aortic arch TBR				
- Mean	**1.07**	**1.06**	**17.09**	**0.71**
- Median	1.08	1.02		
- Standard deviation	0.08	0.11		
- Range	0.92-1.22	0.89-1.39		
Pulmonary arteries TBR				
- Mean	**1.09**	**1.08**	**6.76**	**0.28**
- Median	1.10	1.07		
- Standard deviation	0.11	0.08		
- Range	0.78-1.27	0.87-1.27		
Descending aorta TBR				
- Mean	**1.06**	**1.07**	**23.12**	**0.96**
- Median	1.06	1.04		
- Standard deviation	0.07	0.09		
- Range	0.92-1.23	0.95-1.31		
Abdominal aorta TBR				
- Mean	**1.10**	**1.09**	**16.00**	**0.67**
- Median	1.06	1.07		
- Standard deviation	0.19	0.12		
- Range	0.87-1.64	0.83-1.36		
Glandula parotis sinistra TBR				
- Mean	**1.34**	**1.02**	**5.52**	**0.23**
- Median	1.08	1.01		
- Standard deviation	0.75	0.41		
- Range	0.59-3.32	0.34-2.01		
Glandula parotis dextra TBR				

- Mean	**1.37**	**1.01**	**3.35**	**0.14**
- Median	1.20	0.99		
- Standard deviation	0.78	0.43		
- Range	0.63-3.39	0.39-2.34		
a. iliaca externa sinistra TBR				
- Mean	**1.03**	**1.04**	**13.41**	**0.56**
- Median	1.04	1.04		
- Standard deviation	0.24	0.19		
- Range	0.70-1.76	0.81-1.79		
a. iliaca externa dextra TBR				
- Mean	**0.95**	**1.05**	**3.35**	**0.14**
- Median	0.91	1.02		
- Standard deviation	0.22	0.16		
- Range	0.55-1.31	0.82-1.43		
a. femoralis sinistra TBR				
- Mean	**1.04**	**0.93**	**9.75**	**0.41**
- Median	0.95	0.95		
- Standard deviation	0.25	0.19		
- Range	0.83-1.84	0.59-1.32		
a. femoralis dextra TBR				
- Mean	**1.00**	**0.98**	**18.20**	**0.76**
- Median	0.98	0.96		
- Standard deviation	0.20	0.26		
- Range	0.72-1.46	0.43-1.67		
a. tibialis sinistra TBR				
- Mean	**1.02**	0.93	**13.50**	**0.56**

- Median	0.99	0.96		
- Standard deviation	0.29	0.15		
- Range	0.61-1.86	0.66-1.12		
a. tibialis dextra TBR				
- Mean	**0.99**	0.96	**11.69**	**0.49**
- Median	0.91	0.99		
- Standard deviation	0.29	0.18		
- Range	0.68-1.92	0.59-1.26		
Liver TBR				
- Mean	**1.47**	**1.34**	**0.18**	**0.01**
- Median	1.46	1.33		
- Standard deviation	0.12	0.11		
- Range	1.26-1.66	1.13-1.56		
m. brachioradialis sinistra TBR				
- Mean	**0.49**	**0.48**	**14.59**	**0.61**
- Median	0.44	0.41		
- Standard deviation	0.14	0.23		
- Range	0.35-0.79	0.28-1.25		
m. brachioradialis dextra TBR				
- Mean	**0.49**	**0.47**	**18.20**	**0.76**
- Median	0.40	0.46		
- Standard deviation	0.16	0.11		
- Range	0.31-0.78	0.33-0.75		
